# The Role of Autophagy in the Function of CD4^+^ T Cells and the Development of Chronic Inflammatory Diseases

**DOI:** 10.3389/fphar.2022.860146

**Published:** 2022-03-22

**Authors:** Jiung Jeong, Young Joon Choi, Heung Kyu Lee

**Affiliations:** ^1^ Graduate School of Medical Science and Engineering, Korea Advanced Institute of Science and Technology (KAIST), Daejeon, Korea; ^2^ Department of Internal Medicine, Seoul National University Hospital, Seoul, Korea

**Keywords:** autophagy, CD4+ T cell, asthma, Crohn’s disease, rheumatoid arthritis, multiple sclerosis, systemic lupus erythematosus

## Abstract

Uncontrolled acute inflammation progresses to persistent inflammation that leads to various chronic inflammatory diseases, including asthma, Crohn’s disease, rheumatoid arthritis, multiple sclerosis, and systemic lupus erythematosus. CD4^+^ T cells are key immune cells that determine the development of these chronic inflammatory diseases. CD4^+^ T cells orchestrate adaptive immune responses by producing cytokines and effector molecules. These functional roles of T cells vary depending on the surrounding inflammatory or anatomical environment. Autophagy is an important process that can regulate the function of CD4^+^ T cells. By lysosomal degradation of cytoplasmic materials, autophagy mediates CD4^+^ T cell-mediated immune responses, including cytokine production, proliferation, and differentiation. Furthermore, through canonical processes involving autophagy machinery, autophagy also contributes to the development of chronic inflammatory diseases. Therefore, a targeted intervention of autophagy processes could be used to treat chronic inflammatory diseases. This review focuses on the role of autophagy via CD4^+^ T cells in the pathogenesis and treatment of such diseases. In particular, we explore the underlying mechanisms of autophagy in the regulation of CD4^+^ T cell metabolism, survival, development, proliferation, differentiation, and aging. Furthermore, we suggest that autophagy-mediated modulation of CD4^+^ T cells is a promising therapeutic target for treating chronic inflammatory diseases.

## 1 Introduction

Autophagy is an evolutionarily conserved mechanism in which cytoplasmic materials, such as soluble macromolecules or organelles, are degraded by the lysosome (Matsuzawa-Ishimoto et al., 2018). The main role of autophagy is to maintain cellular homeostasis through starvation adaptation, degradation of misfolded protein and damaged organelles, antiapoptotic function, and elimination of intracellular pathogens (Matsuzawa-Ishimoto et al., 2018).

**FIGURE 1:**
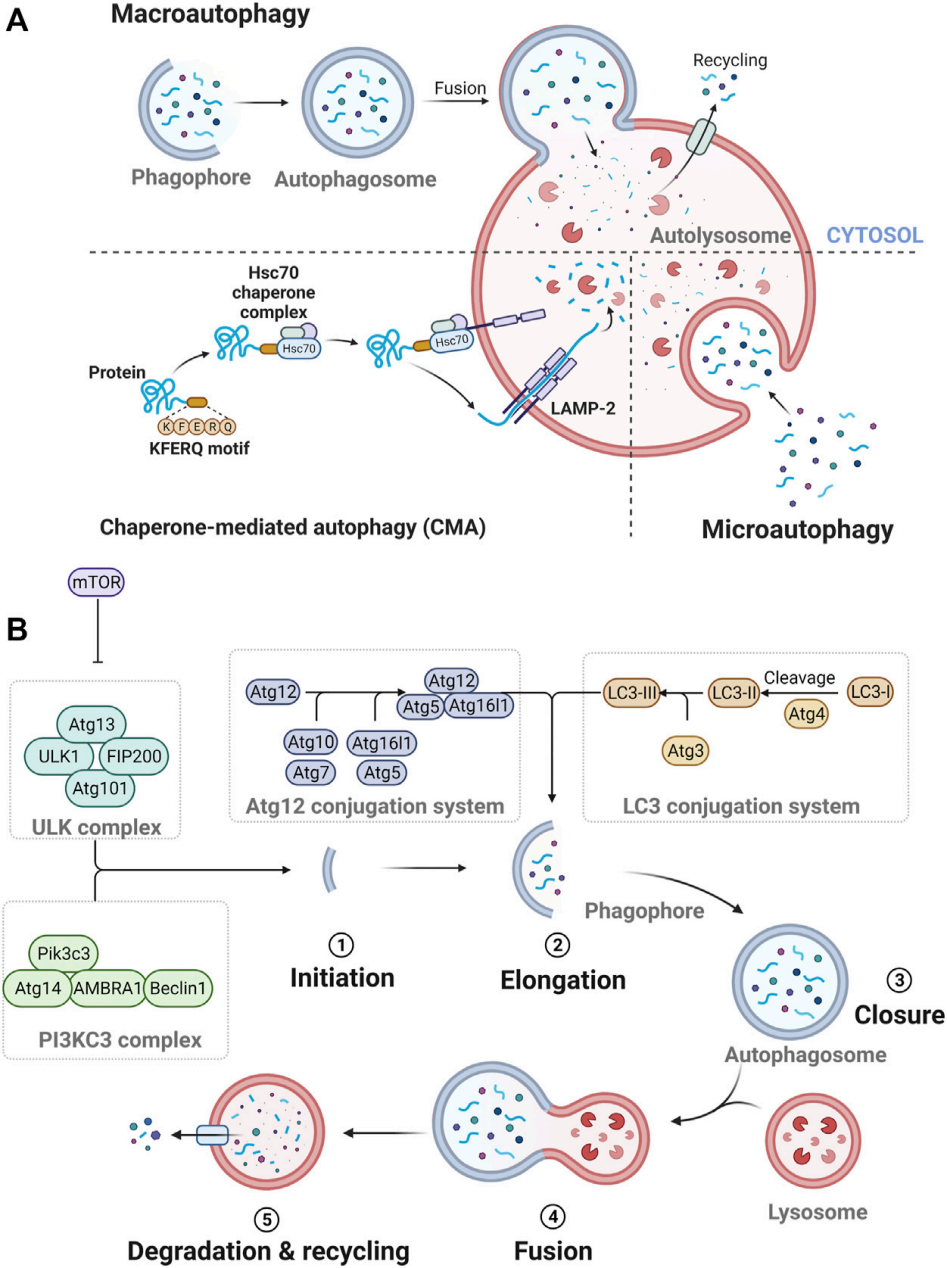
The process of autophagy. **(A)** Three types of autophagy. **(B)** The process of autophagolysosome formation.

Three types of autophagy occur: macroautophagy, microautophagy, and chaperone-mediated autophagy (CMA) (Figure 1A). In macroautophagy, an isolation membrane, termed the phagophore, sequesters a portion of the cytoplasm, resulting in the formation of a double-membrane structure called the autophagosome (Merkley et al., 2018). The autophagosome fuses with the lysosome to become an autolysosome and degrades its cargos with lysosomal enzymes (Merkley et al., 2018). In microautophagy, several components of the cytoplasm are engulfed by inward invagination of the lysosomal membrane (Merkley et al., 2018). In CMA, some specific proteins are recognized by chaperone proteins and translocated across the lysosomal membrane by interacting with lysosome-associated membrane protein type 2A (LAMP-2A) (Merkley et al., 2018).

Specifically, the formation of autophagosome depends on the hierarchically ordered activity of autophagy-related (Atg) proteins (Melia et al., 2020) (Figure 1B). A subset of core Atg proteins is required for autophagosome formation through five sequential steps: initiation, elongation, closure, fusion, and degradation and recycling (Melia et al., 2020). In this pathway, these Atg proteins consist of several functional units: Unc51-like kinase (ULK) complex, class III phosphatidylinositol 3-kinase (PI3KC3) complex, Atg12 conjugation system, microtubule-associated protein 1A/1B-light chain 3 (LC3) conjugation system, and Atg2 complexes (Melia et al., 2020).

Autophagy has a broad relationship to inflammation because their cargos, such as microbes, damaged organelles, and protein aggregates, act as inflammatory signals (e.g., pathogen-associated molecular patterns and danger-associated molecular patterns) (Oh and Lee, 2012; Gomes and Dikic, 2014). In addition, autophagy proteins directly regulate several immune signaling complexes related to type I interferon (IFN) production, inflammasome activation, and antigen presentation (Lee et al., 2007, 2010; Saitoh et al., 2008; Tal et al., 2009). Of the representative targets of autophagy, mitochondria are also the major participants in immunometabolism by regulating cellular metabolism between oxidative phosphorylation and glycolysis, which is important for activation states of macrophages, T cells, and other immune cells (Ip et al., 2017; Mills et al., 2017). Specifically, the multiple “life stages” of CD4^+^ T cells, from development to differentiation, are closely interconnected with dynamic changes in cellular integrity and metabolism. Therefore, autophagy, a critical regulator of cellular metabolism, affects various functions of CD4^+^ T cells, including proliferation, survival, aging, and differentiation (Merkley et al., 2018). In addition, modulation of autophagy in CD4^+^ T cells can relieve the development of chronic inflammatory disease because CD4^+^ T cells are known to be determinants of disease progression.

In this review, we discuss the effect of autophagy on the following functional changes in CD4^+^ T cells: metabolism, survival, development, proliferation, differentiation, and aging. We summarize the phenotypes of T cell-specific autophagy depletion mice in detail (Table 1). Additionally, we investigate whether functional changes in autophagy-induced CD4^+^ T cells can modulate chronic inflammatory diseases.

**TABLE 1:** Phenotypes of T cell-specific autophagy-deficient mice

	Mammalian gene	Mutant mouse	Phenotype	References
ULK complex	*FIP200*	*FIP200* ^*f/f*^ CD4-Cre	T cell apoptosis ↑	Xia et al. (2017)
			Normal thymic cellularity	
			Peripheral T cell number ↓	
		*FIP200* ^*f/f*^ Tie2-Cre	Hematopoietic defect	Liu et al. (2010)
PI3KC3 complex	*Pik3c3*	*Pik3c3* ^*f/f*^ CD4-Cre	T cell apoptosis ↑	Willinger and Flavell, (2012) Parekh et al. (2013) Yang et al. (2021)
			Normal thymic cellularity	
			Peripheral T cell number ↓	
			Th1 differentiation ↓	
			Treg differentiation ↓	
		*Pik3c3* ^*f/f*^ Lck-Cre	T cell apoptosis ↑	McLeod et al. (2011)
			Thymic cellularity ↓	
			Peripheral T cell number ↓	
	*Beclin 1*	*Becn1* ^*f/f*^ CD4-Cre	Peripheral T cell number ↓	Kovacs et al. (2012) Xia et al. (2020)
			Effector to naïve T cell ratio ↑	
			T cell apoptosis ↑	
			Th1 differentiation ↓	
Atg12 conjugation system	*Atg7*	*Atg7* ^*f/f*^ Lck-Cre	T cell apoptosis ↑	Pua et al. (2009) Hubbard et al. (2010) Jia et al. (2011) Amersfoort et al. (2018)
			ER & mitochondria size ↑	
			Peripheral T cell number ↓	
			ATP production ↓	
			Cytokine production ↓	
		*Atg7* ^*f/f*^ CD4-Cre	T cell apoptosis ↑	Lin et al. (2014)
		*Atg7* ^*f/f*^ Foxp3-Cre	T cell apoptosis ↑	Le Texier et al. (2016) Wei et al. (2016)
			Glycolysis ↑	
			Treg differentiation ↓	
	*Atg5*	*Atg5* ^*f/f*^ Lck-Cre	T cell apoptosis ↑	Stephenson et al. (2009) Oravecz-Wilson et al. (2021)
			T cell proliferation ↓	
			Thymic cellularity ↓	
			Peripheral T cell number ↓	
			ER & mitochondria size ↑	
		*Atg5* ^*f/f*^ CD4-Cre	Th9 differentiation ↑	Rivera Vargas et al. (2017)
		*Atg5* ^*f/f*^ Foxp3-Cre	T cell apoptosis ↑	Wei et al. (2016) Plaza-Sirvent et al. (2021)
			Treg differentiation ↓	
LC3 conjugation system	*Atg3*	*Atg3* ^*f/f*^ Lck-Cre	T cell apoptosis ↑	Jia and He, (2011)
			ER & mitochondria size ↑	
		*Atg3* ^*f/f*^ ER-Cre	T cell proliferation ↓	Jia et al. (2015) Rivera Vargas et al. (2017)
			Th9 differentiation ↑	
	*LC3*	*Map1lc3b* ^*−/−*^	Th9 differentiation ↑	Rivera Vargas et al. (2017)

## 2 CD4^+^ T Cell Metabolism

**FIGURE 2:**
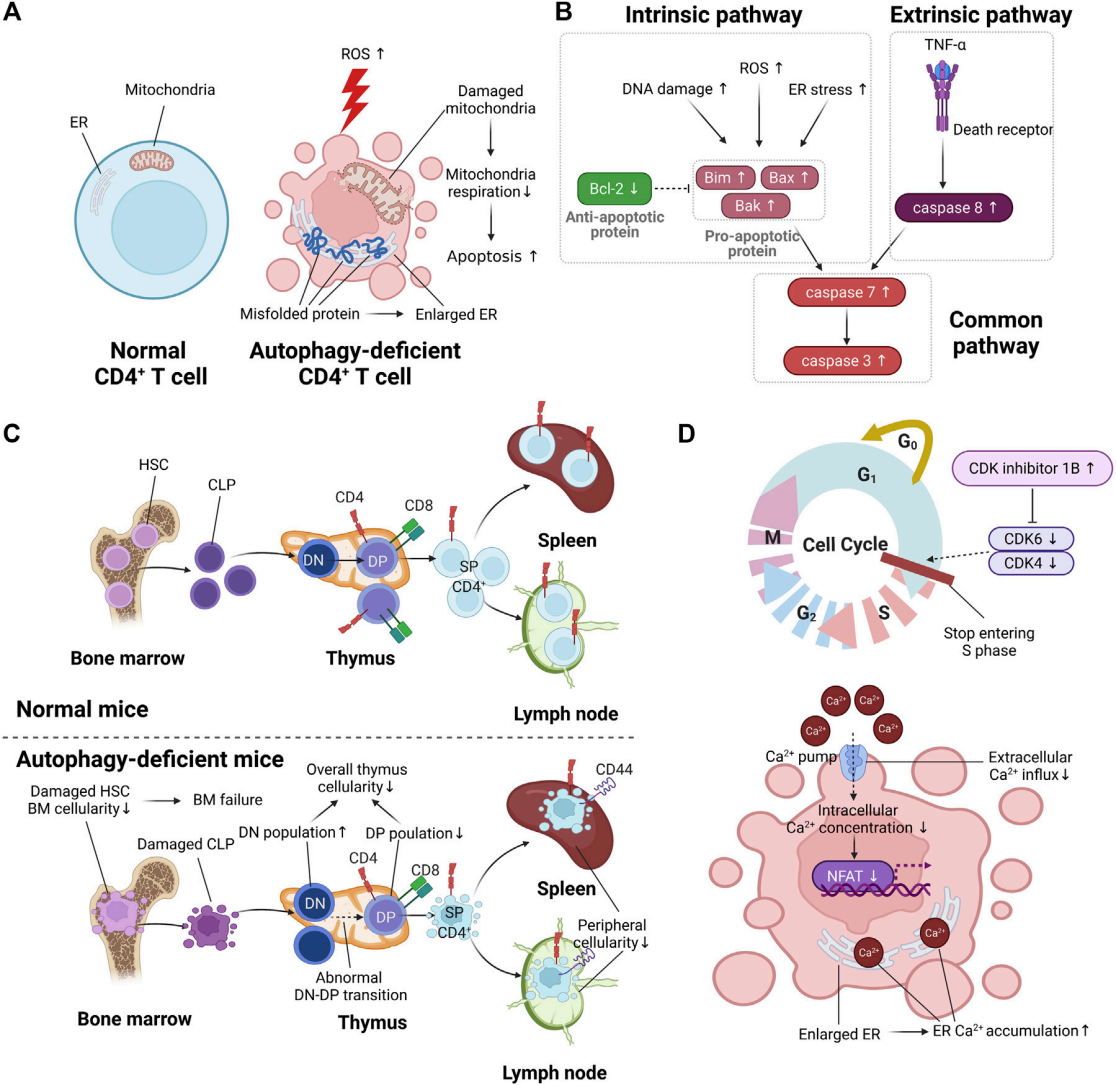
The effect of autophagy on T cell function. **(A)** The difference in metabolic function between normal CD4^+^ T cells and autophagy-deficient CD4^+^ T cells. Autophagy deficiency in CD4^+^ T cells is marked by enlarged endoplasmic reticulum (ER) due to the accumulation of misfolded protein. The increment of reactive oxygen species (ROS) by autophagy deficiency causes damaged mitochondria, resulting in increased apoptosis. **(B)** The increased apoptotic pathway in autophagy-deficient CD4^+^ T cells. **(C)** The difference in CD4^+^ T cell development between normal CD4^+^ T cells and autophagy-deficient CD4^+^ T cells. Autophagy deficiency of hematopoietic stem cells (HSCs) in bone marrow (BM) causes reduced cellularity of BM, resulting in BM failure. Autophagy deficiency in the thymus causes abnormal double-negative (DN)/double-positive (DP) transition, resulting in reduced overall cellularity in the thymus. Autophagy deficiency in peripheral organs such as the spleen and lymph nodes is marked by reduced peripheral cellularity. **(D)** The abnormal cell cycle and reduced intracellular calcium signaling in autophagy-deficient CD4^+^ T cells.

In general, autophagy is known to regulate cellular metabolism under various conditions such as starvation, oxidative stress, and pathogen invasion (Dikic and Elazar, 2018). In these various conditions, autophagy is essential for maintaining cellular homeostasis and obtaining energy for cellular survival (Chun and Kim, 2018). In CD4^+^ T cells, autophagy regulates cellular metabolism and plays a pivotal role in maintaining cellular homeostasis and obtaining energy. In this section, we investigate the effect of autophagy on CD4^+^ T cell metabolism (Figure 2A).

Autophagy is a regulator of reactive oxygen species (ROS), chemically oxygenated reactive molecules such as superoxide, hydrogen peroxide, and hydroxyl radicals (Hayyan et al., 2016). ROS are increased during CD4^+^ T cell activation, and the increased ROS are utilized as a secondary messenger of T cell receptor signaling (Yarosz and Chang, 2018). However, the elevated ROS beyond the permissible range of the intracellular antioxidant system causes T cell death (Rashida Gnanaprakasam et al., 2018). Several studies have revealed that the deficiency of autophagy causes T cell apoptosis by increasing ROS. According to Pua et al., Atg7-deficient murine CD4^+^ T cells exhibit a higher production of ROS than normal CD4^+^ T cells, resulting in increased number of 7-aminoactinomycin D (7-AAD)^+^/Annexin V^+^ apoptotic cells (Pua et al., 2009). The regulation of ROS could reduce autophagy deficient-induced apoptosis. Watanabe reported that the overexpression of peroxiredoxin 1, which is an intracellular H_2_O_2_ scavenger, in ULK-knockdown human CD4^+^ T cells reduced the number of 7-AAD^+^/Annexin V^+^ apoptotic cells (Watanabe et al., 2014) compared with untreated ULK-knockdown human CD4^+^ T cells. These results suggest that autophagy regulates ROS accumulation, resulting in inhibition of apoptosis.

Autophagy is related to the regulation of ATP production in CD4^+^ T cells, which consists of two main steps: (1) glycolysis and (2) oxidative phosphorylation (Palmer et al., 2015). Naïve CD4^+^ T cells acquire ATP from oxidative phosphorylation, and effector CD4^+^ T cells acquire ATP from both aerobic glycolysis and oxidative phosphorylation (Palmer et al., 2015). Deficiency of autophagy results in decreased ATP production. Hubbard et al. reported that autophagy deficiency induced by chemical agents, including ammonium chloride and leupeptin, which are lysosomal proteolysis inhibitors, in murine T cells leads to reduced ATP production (Hubbard et al., 2010). Due to this reduction, decreased production of cytokines, including IFN-γ and IL-2, was observed in autophagy-deficient murine T cells induced by ammonium chloride and leupeptin and in Atg7-deficient CD4^+^ T cells compared with normal CD4^+^ T cells (Hubbard et al., 2010). The regulation of ATP production by autophagy is associated with mitochondrial respiration. According to Mocholi et al., chemically induced autophagy-deficient murine CD4^+^ T cells show reduced mitochondrial respiration, resulting in decreased T cell activation and ATP production (Mocholi et al., 2018). As a result, *Atg7* ^*f/f*^ CD4-Cre mice have a higher survival rate than normal control mice in a myelin oligodendrocyte glycoprotein (MOG)-induced experimental autoimmune encephalomyelitis (EAE) model (Mocholi et al., 2018). These results indicate that autophagy is involved in ATP production through the regulation of mitochondria respiration.

## 3 CD4^+^ T Cell Survival

Autophagy contributes to the maintenance of T cell survival by inhibiting apoptosis. When the pathogen disappears and the inflammatory response is reduced due to adequately activated adaptive immunity, the remaining CD4^+^ T cells undergo apoptosis, except for memory CD4^+^ T cells (Zhan et al., 2017). The pathways involved in apoptosis include the intrinsic pathway and the extrinsic pathway (Galluzzi et al., 2018). Intrinsic apoptosis, caused by DNA damage, ROS, and endoplasmic reticulum (ER) stress, is characterized by upregulation of proapoptotic proteins such as Bim, Bax, and Bak (Galluzzi et al., 2018). The antiapoptotic protein Bcl-2 prevents intrinsic apoptosis by inhibiting oligomerization of Bax and Bak (Brown et al., 2017). Extrinsic apoptosis, characterized by expression of caspase-8, is activated by the binding of TNF-α to a death receptor (Zhan et al., 2017). Both intrinsic and extrinsic apoptosis commonly induce programmed cell death through activation of caspase-3 and caspase-7 (Zhan et al., 2017) (Figure 2B).

As mentioned briefly in the T cell metabolism of the Section 2, autophagy modulates the harmful effects of ROS. Therefore, a deficiency of autophagy leads to programmed cell death by increased ROS. According to Xia et al., ROS accumulation was observed in *in vitro*-activated, FIP200-deficient murine T cells with anti-CD3 and anti-CD28 compared with *in vitro*-activated normal murine T cells, resulting in increased 7-AAD^+^/Annexin V^+^ apoptotic cells (Xia et al., 2017). Abnormalities in FIP200, as well as in other autophagy-related proteins, including Pik3c3, Atg7, and Atg3, in murine CD4^+^ T cells showed increased 7-AAD^+^/Annexin V^+^ apoptotic cells due to ROS accumulation (Pua et al., 2009; Jia and He, 2011; McLeod et al., 2011; Willinger and Flavell, 2012). Similar to what was observed in the murine CD4^+^ T cell-related studies, administration of the autophagy blockers chloroquine and spautin-1 in *in vitro*-activated human CD4^+^ T cells caused increased 7-AAD^+^/Annexin V^+^ apoptotic cells (Watanabe et al., 2014). Therefore, autophagy-deficient mice are vulnerable to infection by external pathogens due to low T cell survival. Lin et al. reported that *Atg7* ^*f/f*^ CD4-Cre mice show higher mortality in a cecal ligation and puncture model due to increased apoptosis in CD4^+^ T cells and CD8^+^ T cells in Atg7-deficient T cells (Lin et al., 2014).

Autophagy regulates the activity of apoptosis-related proteins. The intrinsic apoptotic–related gene is upregulated in autophagy-deficient CD4^+^ T cells. ULK knockdown of human CD4^+^ T cells using a retroviral vector showed the upregulation of *Bim*, which is associated with the intrinsic apoptotic pathway, resulting in increased activation of pan-caspase (Watanabe et al., 2014). Oravecz-Wilson et al. reported that Atg5-deficient murine T cells exhibit lower levels of the antiapoptotic protein Bcl-2 than normal CD4^+^ T cells (Oravecz-Wilson et al., 2021). These results suggest that Bcl-2 expression was decreased due to autophagy deficiency, resulting in increased Bim involved in intrinsic apoptosis. Autophagy deficiency affects not only intrinsic apoptosis but also extrinsic apoptosis. Sequestosome 1 (p62) induces extrinsic apoptosis via caspase-8 activation (Huang et al., 2013). According to Kovacs et al., Beclin 1-deficient murine CD4^+^ T cells show increased caspase-8 activation by protein-protein interaction with p62 (Kovacs et al., 2012). Conversely, inhibition of caspase protein in CD4^+^ T cells reduces the apoptosis of autophagy-deficient CD4^+^ T cells. For example, administration of Q-VD-Oph, a pan-caspase inhibitor, in ULK-knockdown human CD4^+^ T cells resulted in a reduced number of apoptotic human CD4^+^ T cells, similar to normal human CD4^+^ T cells (Watanabe et al., 2014). These results suggest that autophagy inhibits apoptosis by regulating apoptosis-related protein, including caspase proteins.

## 4 CD4^+^ T Cell Development

T cell development consists of three main steps: (1) hematopoiesis in bone marrow, (2) thymic selection in the thymus, and (3) peripheral maintenance in peripheral organ, such as spleen and lymph nodes. Specifically, common lymphoid progenitors (CLPs) are derived from hematopoietic stem cells (HSCs) of bone marrow (Lee et al., 2019). CLPs derived from HSCs migrate to the thymus and mature into CD4^+^ T cells or CD8^+^ T cells through thymic selection (Takaba and Takayanagi, 2017). Mature CD4^+^ T cells or CD8^+^ T cells migrate to peripheral organs and are involved in adaptive immunity (Krummel et al., 2016). Several studies have provided information about the involvement of autophagy in T cell development. In this section, we discuss the effect of autophagy on the three main steps of T cell development (Figure 2C).

### 4.1 Hematopoiesis in Bone Marrow

Autophagy acts as a regulator of hematopoiesis in the bone marrow. For example, FIP200, a component of the ULK-Atg13-FIP200 complex that is related to the initiation of autophagosome, participates in the maintenance and function of HSCs (Liu et al., 2010). Liu et al. reported that FIP200-deficient HSCs exhibit abnormal function due to increased ROS and mitochondrial dysfunction, resulting in hematopoietic defects such as severe erythroblastic anemia and myeloid dysfunction (Liu et al., 2010). Consistent with this finding, *Atg7* ^*f/f*^ Vav-iCre mice showed ROS accumulation and mitochondrial dysfunction in HSCs, resulting in significant multi-lineage cytopenia, including T cells, B cells, NK cells, and myeloid cells (Mortensen et al., 2011). These data indicate that autophagy deficiency in HSCs causes impaired hematopoiesis that results in bone marrow failure.

### 4.2 Thymic Selection

CLPs in the cortex of the thymus are double-negative cells, expressing neither CD4 nor CD8 (Yates, 2014). By T cell receptor rearrangement, double-negative cells are converted to double-positive cells with both CD4 and CD8 (Yates, 2014). Through thymic selection, mature single-positive CD4^+^ T cells or single-positive CD8^+^ T cells are present in the medulla of the thymus (Yates, 2014).

Autophagy is an important factor in determining cellularity in the thymus. Arsov et al. reported that thymic cellularity was reduced in a Beclin 1-deficient, Rag1-knockout (KO) chimera mouse model (Arsov et al., 2011). Subpopulation analysis of thymocytes showed that CD4^−^CD8^−^ double-negative cells were increased, but CD4^+^CD8^+^ double-positive cells were decreased (Arsov et al., 2011). Consistent with this finding, *Pik3c3* ^*f/f*^ Lck-Cre mice and *Atg7* ^*f/f*^ Lck-Cre mice exhibited significant reductions of single-positive CD4^+^ T cells and single-positive CD8^+^ T cells in the thymus (Pua et al., 2009; McLeod et al., 2011). However, *FIP200* ^*f/f*^ CD4-Cre and *Pik3c3* ^*f/f*^ CD4-Cre mice showed normal thymic cellularity (Willinger and Flavell, 2012; Parekh et al., 2013; Xia et al., 2017). The difference between Lck-Cre and CD4-Cre suggests that autophagy affects thymocyte survival and proliferation during the double-negative/double-positive transition.

### 4.3 Peripheral Maintenance

Autophagy is related to maintenance of the peripheral T cell response. According to Pua et al., reduced numbers of CD4^+^ T cells and CD8^+^ T cells were observed in the spleen of *Atg5* ^*−/−*^ chimeric mice (Pua et al., 2007). *Pik3c3* ^*f/f*^ Lck-Cre mice and *Atg7* ^*f/f*^ Lck-Cre mice showed less CD4^+^ T cells and CD8^+^ T cells in the spleen and lymph node than normal control mice (McLeod et al., 2011; Amersfoort et al., 2018). Interestingly, despite normal thymic cellularity, impaired peripheral T cell maintenance was also observed in *FIP200* ^*f/f*^ CD4-Cre and *Pik3c3* ^*f/f*^ CD4-Cre mice (Willinger and Flavell, 2012; Parekh et al., 2013; Xia et al., 2017). These results suggest that regardless of Lck-Cre and CD4-Cre, peripheral T cells with autophagic dysfunction cannot maintain homeostasis, resulting in reduced peripheral cellularity of T cells.

In the analysis of functional properties of peripheral T cells, autophagy-deficient T cells exhibited a memory-like phenotype. Beclin1^f/f^ CD4-Cre mice and *Atg7* ^*f/f*^ Lck-Cre mice showed a high population of CD44^high^ CD62L^low^ peripheral T cells (Pua et al., 2009; Xia et al., 2020). This increase of the memory-like phenotype in autophagy-deficient peripheral T cells might be associated with the host compensating for peripheral lymphopenia.

## 5 CD4^+^ T Cell Proliferation

When CD4^+^ T cells in peripheral organs recognize major histocompatibility complex class II (MHC II)-bound antigen peptide, CD4^+^ T cell proliferation proceeds via T cell receptor (TCR) signaling (Hwang et al., 2020). At this time, autophagy is a determinant of CD4^+^ T cell proliferation. Therefore, the lack of autophagy in CD4^+^ T cells leads to their decreased proliferation. For example, Atg5-deficient murine T cells showed less CD4^+^ T cell proliferation than normal CD4^+^ T cells (Stephenson et al., 2009; Oravecz-Wilson et al., 2021). Additionally, Atg3-deficient murine T cells exhibited less CD4^+^ T cell proliferation than normal CD4^+^ T cells (Jia et al., 2015). Autophagy determines CD4^+^ T cell proliferation because it regulates the cell cycle and intracellular calcium signaling (Figure 2D).

Autophagy plays a central role in maintaining the cell cycle. When naïve CD4^+^ T cells remaining in G0 (quiescent state) become activated, the cell cycle consisting of four phases proceeds: (1) the first gap phase (G1), (2) DNA synthesis phase (S), (3) the second gap phase (G2), and (4) mitosis (M) (Lewis and Ly, 2021). To progress G0/G1 to S, upregulation of cyclin-dependent kinase (CDK)-related genes, including CDK4 and CDK6, is required (Lewis and Ly, 2021). CDK inhibitors prevent excessive T cell proliferation through downregulation of CDK4 and CDK6 (Lewis and Ly, 2021). Jia et al. reported that Atg7-deficient murine CD4^+^ T cells stop entering the S phase of the cell cycle, resulting in decreased proliferation (Jia et al., 2015). The accumulation of CDK inhibitor 1B was observed in Atg7-deficient murine CD4^+^ T cells compared with normal murine CD4^+^ T cells (Jia et al., 2015). These results implicate autophagy in the progression from G0/G1 to S by regulating the degradation of CKD inhibitor 1B.

Autophagy maintains the proliferation of CD4^+^ T cells by regulating intracellular calcium signaling. Taking a closer look at intracellular calcium signaling in activated T cells, phosphorylation of phospholipase C gamma achieved in activated T cells promotes calcium release from the ER to the cytoplasm via inositol 1,4,5-trisphosphate receptor in the ER membrane (Trebak and Kinet, 2019). Reduced concentration of calcium in the ER induces calcium influx from the extracellular space, which greatly increases the intracellular calcium concentration (Trebak and Kinet, 2019). When calcium concentration in the cytoplasm of CD4^+^ T cells is increased through calcium influx, CD4^+^ T cell proliferation–related genes such as nuclear factor of activated T cells (*NFAT*) 1 and *NFAT2* are upregulated (Trebak and Kinet, 2019). The enlarged ER caused by autophagy deficiency increases calcium storage in the ER. Non-degraded proteins due to autophagy deficiency accumulate in the ER and mitochondria. Several studies have reported enlarged ER and mitochondria in autophagy-deficient murine CD4^+^ T cells, including Atg7, Atg5, and Atg3 (Pua et al., 2009; Stephenson et al., 2009; Jia and He, 2011). According to Jia et al., Atg7-deficient murine CD4^+^ T cells exhibited increased calcium concentration in the ER due to enlarged ER (Jia et al., 2011). As calcium influx was relatively reduced due to increased calcium storage in the ER, T cell proliferation was reduced in Atg7-deficient murine CD4^+^ T cells (Jia et al., 2011). Because the amount of exogenous calcium influx (1–2 mM Ca^2+^) is greater than that of calcium release from the ER (300 μM–1 mM Ca^2+^), autophagy-deficient murine CD4^+^ T cells showed reduced CD4^+^ T cell proliferation, despite the enlarged ER (Trebak and Kinet, 2019). Taken together, autophagy deficiency characterized by increased calcium storage in the enlarged ER reduces the calcium influx of high concentration from the extracellular space, resulting in decreased CD4^+^ T cell proliferation due to reduced overall intracellular calcium concentration.

Interestingly, the expression of CD25 and CD69, the early activation markers of T cells, in Atg5-deficient murine T cells is similar to that of normal murine T cells (Stephenson et al., 2009). Although the degree of intracellular calcium signaling is autophagy-dependent, autophagy deficiency does not significantly affect the expression of early activation markers. This result indicates that even if cells are autophagy-deficient, T cell activation proceeds, and autophagy is essential for T cell homeostasis, rather than an antigen-induced expansion signal.

## 6 CD4^+^ T Differentiation

Peripheral CD4^+^ T cells affected by the surrounding microenvironment can be differentiated into various CD4^+^ T cell subsets, including T helper (Th)1, Th2, Th9, and Th17 and regulatory T (Treg) cells (Lee et al., 2021). The effect of autophagy on each CD4^+^ T cell subset is different. For example, autophagy has an inhibitory effect on the differentiation of Th2 and Th9 cells, whereas it promotes the differentiation of Th1 and Treg cells (Merkley et al., 2018). Autophagy affects CD4^+^ T cell subtype differentiation differently possibly due to the changes in metabolic status, the regulation of transcription factor, and the viability of each T cell subset. Below, we examine the effect and mechanism of autophagy on various CD4^+^ T cell subsets in detail.

### 6.1 Regulatory T Cells

**FIGURE 3:**
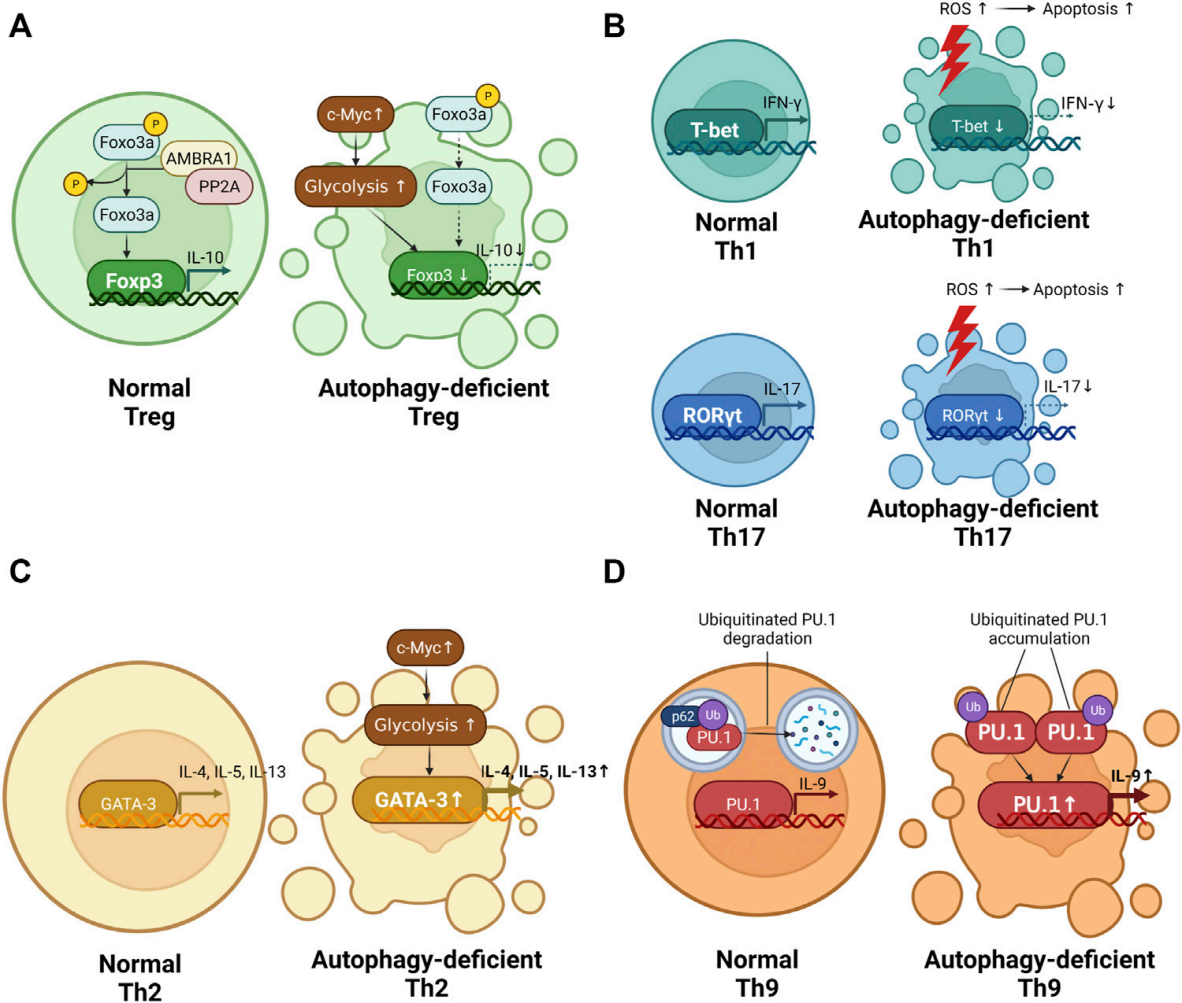
The role of autophagy in T cell differentiation. **(A)** The difference in regulatory T cell (Treg) differentiation between normal CD4^+^ T cells and autophagy-deficient CD4^+^ T cells. Autophagy deficiency in Treg causes reduced interleukin (IL) 10 production. **(B)** The difference in T helper **(**Th)1 and Th17 differentiation between normal CD4^+^ T cells and autophagy-deficient CD4^+^ T cells. Autophagy deficiency in Th1 and Th17 alleviates the production of interferon gamma (IFN-γ) and IL-17. **(C)** The difference in Th2 differentiation between normal CD4^+^ T cells and autophagy-deficient CD4^+^ T cells. Autophagy deficiency in Th2 promotes IL-4, IL-5, and IL-13 production. **(D)** The difference in Th9 differentiation between normal CD4^+^ T cells and autophagy-deficient CD4^+^ T cells. Autophagy deficiency in Th9 promotes IL-9 production.

Treg cells, which express forkhead box P3 (Foxp3) as a transcription factor, suppress the surrounding inflammatory response by secreting IL-10 (Romano et al., 2019). As the only T cell subset that inhibits the inflammatory response, Treg cells have been used to try to treat inflammatory diseases, including asthma and autoimmune disease, as have IL-10 (Coomes et al., 2017; Eggenhuizen et al., 2020). The modulation of autophagy can be used to regulate Treg cell differentiation (Figure 3A).

Autophagy is necessary for maintaining cellularity of Treg cells. Autophagy-deficient mice exhibited a smaller population of Treg cells than normal mice. Parekh et al. reported less Foxp3^+^ Treg cells in the spleen and lymph nodes of *Pik3c3* ^*f/f*^ CD4-Cre mice than in those of normal control mice (Parekh et al., 2013). Consistent with this finding, *Atg5* ^*f/f*^ Foxp3-Cre mice and *Atg7* ^*f/f*^ Foxp3-Cre mice showed less Treg cells in the spleen and lymph nodes than in those of normal control mice (Wei et al., 2016). Autophagy maintains Treg cellularity by affecting the viability of Treg cells, regulation of Foxp3 expression, and changed metabolic status.

As previously described, the function of autophagy in T cells is associated with anti-apoptosis effect. Therefore, autophagy-deficient CD4^+^ T cells show increased apoptosis due to an upregulation of caspase proteins. According to Wei et al., Atg7-or Atg5-deficient murine Treg cells exhibit more active caspase-3 than normal murine Treg cells (Wei et al., 2016). Due to upregulation of active caspase-3, autophagy-deficient Treg cells are susceptible to apoptosis.

Autophagy is involved in Foxp3 induction. The 181–532 amino acids and 946–1269 amino acids of autophagy and Beclin 1 regulator 1 (AMBRA1), a component of C3 complex, form a protein-protein interaction with protein phosphatase 2A (PP2A), which is a Foxp3 inducer by dephosphorylation of Foxo3a (Cianfanelli et al., 2015). According to Becher et al., AMBRA1 knockdown of human CD4^+^ T cells using a lentiviral vector exhibit reduced Foxo3a dephosphorylation, resulting in decreased Foxp3 expression (Becher et al., 2018). As a result, lipopolysaccharide (LPS)-treated AMBRA1^f/f^ Lck-Cre mice exhibited less thymus-derived Treg cells and induced Treg cells than LPS-treated normal control mice (Becher et al., 2018).

Autophagy maintains Treg cell lineages by inhibiting mammalian target of rapamycin (mTOR)-dependent glycolysis. The differentiation of Treg cells relies on fatty acid oxidation rather than glycolysis (Salmond, 2018). *In vitro*-differentiated Treg cells treated with 2-deoxyglucose (2-DG), an inhibitor of glycolysis, showed upregulated Foxp3 expression compared with *in vitro*-differentiated Treg cells treated with the vehicle (Shi et al., 2011). Autophagy deficiency results in reduced Foxp3 expression due to the increased glycolysis. Wei et al. reported that Atg7-deficient murine Treg cells show increased glycolysis compared with normal murine Treg cells (Wei et al., 2016). The increased glycolysis due to autophagy deficiency is related to upregulated c-Myc, an inducer of glycolysis (Wei et al., 2016).

Impaired autophagy in Treg cells causes inflammatory diseases. Inflammatory wasting syndrome, which is characterized by weight loss, intestinal inflammation, and anemia, was observed in aged *Pik3c3* ^*f/f*^ CD4-Cre mice (Parekh et al., 2013). Aged *Atg7* ^*f/f*^ Foxp3-Cre mice had aggravated inflammation in the skin, intestines, and liver compared with same-age normal control mice (Le Texier et al., 2016). Upregulation of autophagy in Treg cells is thought to be useful for the treatment of inflammatory diseases.

### 6.2 Th1 Cells

Th1 cells, which express the T-box protein expressed in T cells (T-bet) as a transcription factor, participate in protecting the host against intracellular bacteria and viruses by secreting IFN-γ (Knochelmann et al., 2018). Autophagy participate in maintaining Th1 differentiation. Autophagy-deficient CD4^+^ T cells shows reduced Th1 differentiation. According to Yang et al., PIK3c3-deficient murine CD4^+^ T cells show less IFN-γ-producing Th1 cells than normal murine CD4^+^ T cells (Yang et al., 2021). In a human CD4^+^ T cell study, the administration of chloroquine, an autophagy blocker, led to reduced production of IFN-γ (Cui et al., 2020). The reasons for autophagy to intervene in Th1 differentiation are the changed survival of Th1 and metabolic status depending on the presence of autophagy.

Autophagy-deficient Th1 cells are vulnerable to cell death (Figure 3B). Kovacs et al. reported that Beclin 1-deficient murine CD4^+^ T cells show reduced Th1 differentiation by increasing apoptosis-related protein such as caspase-3, caspase-8, and Bim (Kovacs et al., 2012). Autophagy is essential for maintaining Th1 survival.

Unfortunately, other studies regarding the effects of autophagy on Th1 differentiation are lacking. Future studies should analyze the mechanism of autophagy in Th1 differentiation in Th1-specific, autophagy-deficient mice generated by mating Tbx21-cre mice.

### 6.3 Th17 Cells

Th17 cells, which express RAR-related orphan receptor gamma as a transcription factor, protect the host against extracellular pathogens and fungi by secreting IL-17 and IL-22 (Knochelmann et al., 2018). Th17 cells are key cells for the development of autoimmune disease including systemic lupus erythematosus (SLE) (Shan et al., 2020). Compared with a healthy control group, the SLE patient group had peripheral blood mononuclear cells (PBMCs) with increased IL-17-producing Th17 cells, resulting in a positive correlation with disease severity (Liu and Wang, 2014).

Autophagy contributes to the induction of Th17 differentiation (Figure 3B). Autophagy deficiency caused reduced Th17 response. Wang et al. reported that the use of bazedoxifene, a regulator of autophagy, reduced IL-17-producing Th17 cells in an experimental autoimmune myocarditis murine model (Wang et al., 2021). In a PBMC-derived human CD4^+^ T cell study, chloroquine-treated PBMCs of SLE patients exhibited a lower Th17 response than SLE patients with untreated PBMCs (An et al., 2017). Similar to Th1 cells, autophagy-deficient Th17 cells are susceptible to cell death. According to Huang et al., autophagy-deficient human CD4^+^ T cells overexpressing microRNA-590-3p showed reduction of Th17 differentiation by increased apoptosis (Huang et al., 2021).

The regulation of Th17 cells by modulating autophagy has been proven to be effective in various mouse disease models. An et al. reported that the administration of autophagic inhibitor hydroxychloroquine to MRL/lpr mice, which spontaneously develop SLE-like inflammation, ameliorates SLE-induced nephritis pathology, such as mesangial cellular proliferation (An et al., 2017). Consistent with this finding, the administration of an microRNA-590-3p agomir to MRL/lpr mice attenuated the pathology score of lupus nephritis (Huang et al., 2021). In a murine autoimmune myocarditis model, the administration of bazedoxifene reduced Th17 differentiation, resulting in improved myocarditis pathology (Wang et al., 2021).

### 6.4 Th2 Cells

Th2 cells, which express GATA binding protein 3 (GATA-3) as a transcription factor, play a role in protecting the host against parasites by secreting IL-4, IL-5, and IL-13 (Knochelmann et al., 2018). Autophagy is known to inhibit Th2 differentiation (Figure 3C). Autophagy deficiency promotes the differentiation of Th2 cells. Kabat et al. reported that *Atg16l1* ^*f/f*^ CD4-cre mice show an increased number of colonic Th2 cells expressing GATA-3 and IL-13 (Kabat et al., 2016). This increase may be related to the reduced number of Foxp3^+^ Treg cells (Kabat et al., 2016). However, the explanation of increased Th2 by reduced Treg cells is difficult to be compatible with the decrease in Th1 and Th17 cells in Atg16l1-deficient mice. Additionally, Kabat et al. paid attention to metabolic changes caused by autophagy deficiency. The effector function of Th2 cells is dependent on glycolysis (Salmond, 2018). Compared with normal control T cells, Atg16l1-deficient T cells showed upregulated glycolytic genes including *GLUT1*, *Slc16a3*, *Tpi1*, *Ldha*, *Aldoa*, *Gpi1*, and *Pgk1* (Kabat et al., 2016). As mentioned briefly in the 6.1. Treg cells section, the upregulation of c-Myc by autophagy deficiency promotes glycolysis (Wei et al., 2016). These results suggest that autophagy deficiency leads to increased glycolysis, resulting in upregulation of GATA-3.

### 6.5 Th9 Cells

Th9 cells, which express PU.1 as a transcription factor, secrete IL-9 (Angkasekwinai and Dong, 2021). Th9 cells are important for regulating allergic disease and cancer (Angkasekwinai and Dong, 2021). Autophagy suppresses the differentiation of Th9 cells (Figure 3D). According to Rivera Vargas et al., *in vitro*-differentiated Atg5-, Atg3-, and LC3-deficient murine Th9 cells exhibited higher IL-9 production than *in vitro*-differentiated normal murine Th9 cells (Rivera Vargas et al., 2017). In addition, *in vitro*-differentiated murine Th9 cells treated with chloroquine showed an increased amount of IL-9 in a dose-dependent manner (Rivera Vargas et al., 2017). The reason that autophagy deficiency increases Th9 differentiation is related to the ubiquitination of PU.1. Ubiquitinated PU.1 was degraded by p62-dependent autophagy (Rivera Vargas et al., 2017). However, PU.1 degradation was reduced in Atg5-deficient murine CD4^+^ T cells compared with normal murine CD4^+^ T cells (Rivera Vargas et al., 2017). Taken together, Atg5-deficient CD4^+^ T cells promote Th9 differentiation by upregulating PU.1.

## 7 CD4^+^ T Cell Aging

Aging is defined as a phenomenon by which the structure and function of the body gradually deteriorates due to cellular damage with the passage of time (Barbosa et al., 2019). Dysregulation of cellular metabolism, including starvation, oxidative stress, and pathogen invasion, aggravate cellular damage, resulting in acceleration of the aging process (Moltedo et al., 2019). Thus, autophagy, which maintains the homeostasis of cellular metabolism, is closely related to the aging process. Malfunction of autophagy causes increased cellular damage, resulting in age-related diseases including neurodegeneration, heart disorders, and cancers (Barbosa et al., 2019).

Several studies have reported that aged mice show reduced autophagic function in various cells including nerve, heart, and muscle cells (Kaushik et al., 2012; García-Prat et al., 2016; Liang et al., 2020). Like other cells, senescent CD4^+^ T cells show impaired autophagic function. According to Valdor et al., 22-month-old mice have in CD4^+^ T cells with lower expression of LAMP-2A, a lysosomal receptor from CMA, than 4-month-old mice (Valdor et al., 2014). In a PBMC-derived human T cell study, T cells from old healthy donors showed reduced autophagic function compared with T cells from young healthy donors (Phadwal et al., 2012).

This reduced autophagic activity in senescent T cells worsens the aging process. Usually, the length of telomeres is used as a parameter to judge cellular senescence (Vaiserman and Krasnienkov, 2021). Accelerated telomere shortening, which indicates cellular senescence is worsening, is often found in patients with age-related diseases (Armanios, 2013). ROS caused by autophagy deficiency shorten the telomeres of T cells. Sanderson et al. reported that PBMC-derived T cells from old healthy donors have greater ROS levels than those from young healthy donors (Sanderson and Simon, 2017). However, the administration of N-acetyl cysteine (NAC), a scavenger of ROS, in PBMC-derived T cells rescued telomere length compared with nontreated PBMC-derived T cells (Sanderson and Simon, 2017). These results indicate that ROS, which is regulated by autophagy, determine T cell senescence by controlling telomere length.

Dysregulated immune reaction due to T cell senescence can be improved by enhancement of autophagy. According to Fernández et al., Beclin 1 knock-in mice show reduced cardiac fibrosis and renal fibrosis compared with normal control mice, resulting in longer survival (Fernández et al., 2018). In human study of adults older than 65 years, those treated with the mTOR inhibitor RAD001 had higher antibody titers against influenza vaccine than the placebo-treated group (Mannick et al., 2018). These results suggest that an autophagy enhancer can be used to regulate abnormal T cell responses in the context of aging. However, because these results were obtained by regulating autophagy in whole-body cells, further research using T cell–specific mice is required.

## 8 Chronic Inflammatory Diseases

Chronic inflammatory diseases, including asthma, Crohn’s disease (CD), rheumatoid arthritis (RA), multiple sclerosis (MS), and SLE, are pathological conditions in which inflammation progresses for several months to several years, resulting in disability of various organs, including the lungs, gastrointestinal tract, and joints (Furman et al., 2019). Autophagy influences the development of such diseases. For example, autophagy is an exacerbating factor in the development of asthma, RA, MS, and SLE but a relieving factor in CD. In this section, we examine the role of autophagy in the development of each chronic inflammatory disease and the corresponding therapeutic potential of regulating autophagy.

### 8.1 Asthma

Asthma is a chronic airway inflammatory disease caused by allergens, including house dust mite (HDM), pollen, and animal dander (Jeong and Lee, 2021). Patients with asthma exposed to allergens complain of respiratory symptoms such as cough, dyspnea, and wheezing due to airway stenosis (Jeong and Lee, 2021). The lung tissue of asthma patients is characterized by mucus secretion via goblet cell hyperplasia and the infiltration of various immune cells, including eosinophils and neutrophils (Jeong and Lee, 2021).

An atypical upregulation of autophagy is observed in asthma patients. Liu et al. reported that the expression of LC3 and autophagosome was increased in bronchoalveolar lavage fluid of ovalbumin-treated mice compared with saline-treated mice (Liu et al., 2016). In human studies, *Atg5* expression was upregulated in the nasal epithelium of asthma patients with acute exacerbation compared with stable asthma patients (Martin et al., 2012). Patients with asthma had higher levels of autophagy-related protein including Beclin 1 and Atg5 in lung tissue than healthy participants (McAlinden et al., 2019). In addition, asthma patients with a genetic variant in *Atg5* (SNP rs12212740 G > A of *Atg5*) from peripheral blood samples showed aggravated lung function compared with other asthma patients without this genetic variant (Poon et al., 2012).

Autophagy plays a role in exacerbating the development of asthma. Sweeter et al. reported that Atg16 hypomorphic mice with IL-33-induced airway inflammation have decreased goblet cell hyperplasia (Sweeter et al., 2021). Consistent with this finding, LC3-deficient mice showed a reduction in HDM-induced airway inflammation and reduced mucus secretion (Li et al., 2020). Therefore, autophagy blockers can be used to improve asthmatic inflammation. In the HDM-induced mouse model, the administration of chloroquine alleviated airway inflammation and improved airway stenosis (McAlinden et al., 2019). The intravenous injection of P140, a downregulator of CMA, attenuated ovalbumin-induced eosinophilic inflammation (Daubeuf et al., 2021).

The asthma-related murine model using ovalbumin and HDM is mainly related to Th2-mediated eosinophilic inflammation. As formerly mentioned in the 6.4. Th2 cells section, upregulated Th2 differentiation was observed in autophagy-deficient mice (Kabat et al., 2016). However, two studies have showed that autophagy deficiency in ovalbumin- or HDM-induced inflammation results in reduced eosinophilic inflammation (McAlinden et al., 2019; Daubeuf et al., 2021). The results of these two studies are the opposite of those expected by Kabat et al. Thus, because these two studies used whole-body autophagy-knockout mice, the specific effect of autophagy on CD4^+^ T cells in asthma development cannot be fully understood. In addition, conflicting results might have been caused by other autophagy-deficient immune cells. To clearly resolve these conflicting results, asthma studies using CD4^+^ T cell–specific autophagy depletion mice are required in the future.

### 8.2 Crohn’s Disease

CD is a chronic inflammatory bowel disease that occurs throughout the gastrointestinal tract from mouth to anus (Roda et al., 2020). Patients with CD experience abdominal pain, gastrointestinal bleeding, diarrhea, and weight loss (Roda et al., 2020). In studies of CD pathology in these patients, Treg cells in the lamina propria are decreased, whereas other T cell subsets, including Th1, Th2, and Th17, are increased, which causes inflammation in gastrointestinal tract (Clough et al., 2020).

Although the pathogenic factors of CD are not well known, a genetic mutation in *Atg16l1* is strongly associated with the development of CD. Hampe et al. reported that *Atg16l1* variants (rs2241880, Thr300Ala) increase the risk of CD development (Hampe et al., 2007). According to Murthy et al., *Atg16l1* variants (rs2241880, Thr300Ala) in murine macrophage and human B cell showed reduced autophagic function (Murthy et al., 2014). In a murine study, *Atg16l1* T300A variant knock-in mice were vulnerable to salmonella-induced cecal inflammation compared with normal mice (Lassen et al., 2014).

Aggravation of CD by genetic mutation of *Atg16l1* is related to the changed population of Treg cells. Kabat et al. reported that Atg16l1-deficient CD4^+^ T cells show a reduced population of Treg cells (Kabat et al., 2016). Aged *Atg16l1* ^*f/f*^ CD4-Cre mice and aged *Atg5* ^*f/f*^ Foxp3-Cre mice showed increased inflammation in the colon and greater weight loss compared with normal control mice (Kabat et al., 2016; Plaza-Sirvent et al., 2021).

Modified autophagy can control the development of CD. L-arginine, a precursor of spermidine, enhances Treg cell differentiation by the induction of IL-10 promoter DNA hypomethylation (Yu et al., 2017). According to Carriche et al., the administration of spermidine in CD4^+^ T cells can induce the differentiation of Treg cells in an autophagy-dependent manner (Carriche et al., 2021). As a result, mice fed a spermidine-rich diet showed improved inflammation in the colon compared with mice fed a normal diet in a T cell transfer model of colitis (Carriche et al., 2021).

### 8.3 Rheumatoid Arthritis

RA is a chronic inflammatory joint disease characterized by infiltration of autoantibodies such as rheumatoid factor and anti-citrullinated protein antibodies (Smolen et al., 2018). Histological features of RA are synovial hyperplasia and the infiltration of various immune cells in articular cartilage (Smolen et al., 2018). Autophagy plays a role in exacerbating the development of RA.

Autophagy-related proteins are increased in patients with RA. Peeters et al. reported that the RNA levels of autophagy-related genes is upregulated in synovial fluid–derived T cells from patients with juvenile RA compared with PBMC-derived T cells from healthy participants (Peeters et al., 2017). In a protein study as well as an RNA study, patients with RA had synovial tissue with higher levels of autophagic protein including Beclin1 and LC3 than patients with osteoarthritis, resulting in reduced apoptosis measured with the TUNEL assay (Xu et al., 2013). In an analysis of PBMC-derived T cells, patients with RA had a higher level of autophagosome formation than healthy participants, resulting in increased resistance to apoptosis (van Loosdregt et al., 2016). These results offer a possibility that the increment of autophagy in CD4^+^ T cells from patients with RA might induce chronic inflammation with upregulated viability of auto-reactive T cells due to reduced apoptotic CD4^+^ T cells.

The inhibition of autophagy can relieve the development of RA. Kim et al. reported that the administration of hydroxychloroquine, a drug used to treat patients with RA, in PBMC-derived human CD4^+^ T cells inhibits autophagic flux by interfering with mitochondrial superoxide production and induces excessive ROS, thereby inhibiting the proliferation of activated CD4^+^ T cells (Kim et al., 2021). According to van Loosdregt et al., the administration of hydroxychloroquine in PBMC-derived human CD4^+^ T cells increases apoptotic CD4^+^ T cells, suggesting hydroxychloroquine improves joint inflammation by reducing auto-reactive T cells (Van Loosdregt et al., 2013).

### 8.4 Multiple Sclerosis

MS is a chronic inflammatory disease characterized by demyelination of the central nervous system (Longo et al., 2018). Auto-reactive CD4^+^ T cells targeting the myelin sheath contribute to the development of MS (Wagner et al., 2020). Due to demyelination of the central nervous system, patients with MS experience various neurologic dysfunctions, including visual loss, limb weakness, sensory loss, and ataxia (Longo et al., 2018).

Increased autophagic proteins are observed in the tissues of MS patients. From the analysis of postmortem brain tissue, the MS patient group showed higher Atg5 protein levels than the healthy control group (Alirezaei et al., 2009). Similarly, PBMC-derived T cells from MS patients showed higher Atg5 protein levels and greater autophagic function than those of healthy participants (Paunovic et al., 2018). As mentioned in Sections 6.2, 6.3, the increment of autophagy in CD4^+^ T cells of MS patients suppresses the apoptosis of Th1 and Th17 cells, resulting in the generation of auto-reactive CD4^+^ T cells.

Inhibiting autophagy in CD4^+^ T cells helps alleviate the symptoms of MS. In an MOG-induced EAE model, abnormal gait and quadriplegia were not observed in *Becn1* ^*f/f*^ CD4-Cre mice compared to control mice (Kovacs et al., 2012). In addition, *Pik3c3* ^*f/f*^ CD4-Cre mice and *Pik3c3* ^*f/f*^ Rosa26-CreER^T2^ mice showed improved clinical scores than control mice (Yang et al., 2021). These results indicate that autophagy might act as an exacerbating factor for MS. Based on these results, several murine experiments for the use of autophagy blockers as a treatment for MS have been ongoing. The administration of chloroquine in MOG-induced EAE mice showed ameliorated clinical score and improved disease-free survival (Bhattacharya et al., 2014). SAR405, a selective PIK3C inhibitor, also reduced the development of MOG-induced EAE (Yang et al., 2020). We expect that autophagy blockers will be used to improve clinical symptoms of MS patients through various clinical trials in the future.

### 8.5 Systemic Lupus Erythematosus

SLE is a chronic inflammatory disease with systemic involvement by autoantibodies, such as anti–double stranded DNA, antiphospholipid, and anti-Smith (Tsokos, 2011). Auto-reactive Th17 cells are involved in the pathogenesis of SLE (Tsokos et al., 2016). Auto-reactive Th17 cells promote neutrophil infiltration by secreting IL-17 and inducing B cells to produce autoantibodies, resulting in skin rash, oral ulcers, kidney disorder, blood disorder, and neurologic disorder (Tsokos et al., 2016).

Autophagy is related to the development of SLE. López et al. reported that an Atg5 mutation (Atg5 rs573775) in PBMCs is a high-risk factor for the development of SLE (López et al., 2013). Increased autophagic vacuoles were observed in the PBMC-derived T cells from SLE patients compared to PBMC-derived T cells from healthy controls (Gros et al., 2012). According to An et al., increased autophagy in CD4 T cells exacerbates lupus nephritis by upregulation of Th17 differentiation and downregulation of Treg differentiation (An et al., 2017).

Hydroxychloroquine is widely used as a treatment for SLE patients (Schrezenmeier and Dörner, 2020). Two possible mechanisms can explain how hydroxychloroquine relieves symptoms of SLE. First, hydroxychloroquine reduces the secretion of proinflammatory cytokines by disrupting endosome TLR7 and TLR9 signaling (Schrezenmeier and Dörner, 2020). Second, hydroxychloroquine, an autophagy blocker, suppresses unnecessary autoantigen presentation by inhibiting excessive lysosomal activity (Schrezenmeier and Dörner, 2020). Therefore, patients with SLE treated with hydroxychloroquine had less activation of PBMC-derived T cells than those without, suggesting that hydroxychloroquine reduces the activation of auto-reactive CD4^+^ T cells (Wu et al., 2017).

Interestingly, Lai et al. reported that the use of sirolimus, an mTOR inhibitor, helped improve symptoms of SLE patients and reduced the daily dose of prednisone (Lai et al., 2018). Sirolimus functions as an autophagy inducer but is mainly used as an immunosuppressant to inhibit T cell proliferation and induce T cell anergy (Stallone et al., 2016). The symptom relief of SLE patients by sirolimus may be due to its immunosuppressant function inhibiting T cell effector functions. In addition, the administration of sirolimus in CD4^+^ T cells may alter the fate of CD4^+^ T cell differentiation. In particular, mTOR suppresses the expression of Foxp3 by antagonizing the function of SMAD3 and SMAD4 (Chi, 2012). Conversely, Th1, Th2, and Th17 differentiation are promoted by mTOR signaling (Chi, 2012). Kato and Perl reported that the administration of sirolimus in CD4^+^ T cells led to an enhanced Treg population and reduced Th17 population (Kato and Perl, 2014). Consistent with this finding, PBMC-derived CD4^+^ T cells treated with sirolimus for 12 months exhibited more Foxp3^+^ Tregs and reduced IL-17 production than at baseline (Lai et al., 2018). These results suggest that sirolimus, as an mTOR inhibitor, might be directly involved in T cell differentiation to promote Treg differentiation and induce symptom relief of SLE by inhibiting Th17 differentiation.

## 9 Conclusion

CD4^+^ T cells act as a conductor to orchestrate surrounding immune cells, including CD8^+^ T cells, macrophages, eosinophils, and neutrophils, by secreting cytokines (Ruterbusch et al., 2020). Depending on the functional change of CD4^+^ T cells, the response of surrounding immune cells can differ (Ruterbusch et al., 2020). Autophagy is an important biological phenomenon that regulates CD4^+^ T cell function. Our literature review identifies the effects of autophagy on CD4^+^ T cell function including metabolism, survival, development, proliferation, differentiation, and aging. In addition, we find that the dysfunction of CD4^+^ T cells caused by autophagy deficiency affects disease progression in humans, as well as in mice.

Chronic inflammatory diseases currently account for more than 50% of deaths in the world (Furman et al., 2019). CD4^+^ T cells have a profound effect on the development of chronic inflammatory diseases (Muehling et al., 2017; Imam et al., 2018; Chemin et al., 2019). Through the regulation of CD4^+^ T cells, therapies are expected to be able to control the progression of chronic inflammatory diseases. However, current available drugs have many limitations, such as efficacy problems and limited coverages, that have not yet been resolved for the treatment of chronic inflammatory diseases by regulating CD4^+^ T cells. As mentioned above, autophagy can control various functions of T cells. In addition, the autophagy blockers, chloroquine and hydroxychloroquine, and a high spermidine diet can relieve chronic inflammation induced by asthma, RA, MS, and SLE. These autophagy blockers may improve disease symptoms using not only an autophagy-dependent process but also an autophagy-independent process (Galluzzi and Green, 2019). The details of the autophagy-independent process will be discussed in a future article. We believe that the modification of autophagy in CD4^+^ T cells could improve the prognosis of patients with chronic inflammatory disease.
